# KSHV vIL-6 enhances inflammatory responses by epigenetic reprogramming

**DOI:** 10.1371/journal.ppat.1011771

**Published:** 2023-11-07

**Authors:** Tomoki Inagaki, Kang-Hsin Wang, Ashish Kumar, Chie Izumiya, Hiroki Miura, Somayeh Komaki, Ryan R. Davis, Clifford G. Tepper, Harutaka Katano, Michiko Shimoda, Yoshihiro Izumiya

**Affiliations:** 1 Department of Dermatology, School of Medicine, the University of California Davis (UC Davis), Sacramento, California, United States of America; 2 Department of Pathology and Laboratory Medicine, School of Medicine, UC Davis, Sacramento, California, United States of America; 3 Department of Biochemistry and Molecular Medicine, School of Medicine, UC Davis, Sacramento, California, United States of America; 4 Department of Pathology, National Institute of Infectious Diseases, Shinjuku, Tokyo, Japan; University of Utah, UNITED STATES

## Abstract

Kaposi sarcoma-associated herpesvirus (KSHV) inflammatory cytokine syndrome (KICS) is a newly described chronic inflammatory disease condition caused by KSHV infection and is characterized by high KSHV viral load and sustained elevations of serum KSHV-encoded IL-6 (vIL-6) and human IL-6 (hIL-6). KICS has significant immortality and greater risks of other complications, including malignancies. Although prolonged inflammatory vIL-6 exposure by persistent KSHV infection is expected to have key roles in subsequent disease development, the biological effects of prolonged vIL-6 exposure remain elusive. Using thiol(SH)-linked alkylation for the metabolic (SLAM) sequencing and Cleavage Under Target & Release Using Nuclease analysis (CUT&RUN), we studied the effect of prolonged vIL-6 exposure in chromatin landscape and resulting cytokine production. The studies showed that prolonged vIL-6 exposure increased Bromodomain containing 4 (BRD4) and histone H3 lysine 27 acetylation co-occupancies on chromatin, and the recruitment sites were frequently co-localized with poised RNA polymerase II with associated enzymes. Increased BRD4 recruitment on promoters was associated with increased and prolonged NF-κB p65 binding after the lipopolysaccharide stimulation. The p65 binding resulted in quicker and sustained transcription bursts from the promoters; this mechanism increased total amounts of hIL-6 and IL-10 in tissue culture. Pretreatment with the BRD4 inhibitors, OTX015 and MZ1, eliminated the enhanced inflammatory cytokine production. These findings suggest that persistent vIL-6 exposure may establish a chromatin landscape favorable for the reactivation of inflammatory responses in monocytes. This epigenetic memory may explain the greater risk of chronic inflammatory disease development in KSHV-infected individuals.

## Author summary

Combined and continuous cytokine stimulation triggers transcription reprogramming and is often used for specific tissue development. Continuous vIL-6 exposure occurs in KSHV-infected patients and leads to inflammatory cytokine storm with high mortality. However, possible epigenetic reprogramming by the vIL-6 and its association with pathogenesis remain unclear. Here, we demonstrate the establishment of a new chromatin landscape mediated by BRD4 through prolonged vIL-6 exposure, contributing to more robust and rapid transcription and increased cytokine production. Inhibition of BRD4 suppressed this inflammatory response. Our results indicate that targeting the epigenetic effect of viral cytokines may lead to novel therapies for KSHV-induced inflammatory cytokine storms.

## Introduction

Controlled inflammatory response enhances host immunity against external insults, whereas uncontrolled inflammatory responses resulting from excessive cytokine production lead to disruption of such balance. In particular, monocytes and macrophages play major roles in controlling pro-inflammatory cytokines expression, and their tightly controlled activation is critical for regulating systemic inflammation in our body. Previous reports have shown that continuous inflammatory stimulation enhances [1,2] or sometimes impairs responses to subsequent stimuli. In either case, continuous inflammatory stimulation appears to alter the cellular phenotype and affect inflammatory cytokine production.

Kaposi’s sarcoma-associated herpesvirus (KSHV) was first identified in Kaposi’s sarcoma (KS) lesions in 1994 [3]. KSHV is also associated with lymphoproliferative diseases, primary effusion lymphoma (PEL), and multicentric Castleman’s disease (MCD) [4,5]. Recent studies showed that KSHV infection also causes severe systemic inflammation, categorized as Kaposi’s sarcoma-associated herpesvirus inflammatory cytokine syndrome (KICS) [6]. KICS is characterized by increased viral loads and inflammatory cytokines such as human IL-6 (hIL-6), IL-10, and KSHV-encoded interleukin-6 homolog, viral IL-6 (vIL-6). Individuals with KICS are known to have a higher risk of developing KSHV-associated cancers and other malignancies [6].

The vIL-6, encoded by KSHV ORF-K2, is expressed during the lytic replication phase, and single-cell transcriptomics studies also suggested that vIL-6 may be expressed during latency in selected cell populations [7]. Similar to hIL-6, vIL-6 is known to enhance cell proliferation, endothelial cell migration, and angiogenesis by upregulating vascular endothelial growth factor (VEGF) [8] and downregulating the caveolin 1 expression [9–11]. Moreover, vIL-6 transduction also leads to cell transformation in the 3T3 cells model and induces tumors in xenograft mice [8]. Increased metastasis is seen in a murine xenograft model with transgenic vIL-6 mice [12]. The vIL-6 activates downstream signaling pathways by binding to the cellular hIL-6 receptor, gp130. Dimerization of gp130 induced by hIL-6 binding activates Janus tyrosine kinases and phosphorylates the SH2-containing cytoplasmic protein STAT3 (signal transducer and activator of transcription 3). The phosphorylated STAT3 forms a dimer and translocates to the nucleus to activate the downstream genes that are essential in inducing the inflammatory response, cell survival, and immune responses [13–15].

Synergistic interactions between another inflammatory-associated transcription factor, nuclear factor-kappa B (NF-κB), and STAT3 induce the hyperactivation of NF-κB, followed by the production of various inflammatory cytokines, including TNFα and hIL-6. Because the hIL-6 expression is regulated by NF-κB pathway activation, simultaneous activation of NF-κB and STAT3 triggers a positive feedback loop of NF-κB activation in the hIL-6/STAT3 axis. This positive feedback loop is called the IL-6 amplifier (IL-6 Amp) and is a critical element in inflammatory disease development [16]. In the IL-6 Amp disease model, obesity, injury, and infection trigger chronic inflammation and a systemic cytokine storm, which is enhanced by interactions between local non-immune cells and infiltrating immune cells [16]. A prominent example of IL-6 Amp is also seen in Coronavirus disease 2019 (COVID-19), which is triggered by severe acute respiratory syndrome coronavirus 2 (SARS-CoV2) infection [16]. Consistent with the significance of hIL-6 in disease development, a humanized monoclonal antibody against the hIL-6 receptor, tocilizumab, has proven successful in treating Castleman’s disease as well as COVID-19 [17,18]. In the case of KSHV-associated diseases, the IL-6 Amp may be triggered by KSHV infection and is likely to be associated with vIL-6 expression from KSHV-infected cells. However, how persistent vIL-6 exposure alters cellular responses and enhances inflammation cytokine production remains elusive.

The bromodomain and extraterminal domain (BET) family proteins, such as bromodomain containing 4 (BRD4), are transcription regulators that localize to cellular enhancer and promoter loci and control the transcription of a wide range of proinflammatory genes. BRD4 regulates transcription by recognizing acetylated histone tail and interacts with transcription factors such as NF-κB p65 and transcription elongation complex [19]. This mechanism facilitates the phosphorylation of RNA Polymerase II (RNAPII) and promotes transcription initiation and elongation. In the context of proinflammatory gene activation, interaction between BRD4 and NF-κB p65 is especially important, because p65 itself is acetylated at K218, K221, and K310, and the BRD4 recognizes the acetylated K310 to form an active transcription complex [20]. In addition to the specific protein-protein interactions, BRD4 also forms nuclear puncta on super-enhancer genomic regions that exhibit properties of liquid-like condensates [21]. The intrinsically disordered regions (IDRs) of BRD4 are responsible for phase-separated condensates formation with other coactivators on the chromatin loci for transcription elongation [21], which suggests that enrichment of BRD4 at specific enhancers or promoters is key for selection of genes for activation.

Here, we demonstrate that prolonged vIL-6 exposures remodel chromatin landscape. We show that an increased amount of BRD4 on chromatin by prolonged vIL-6 exposure leads to transcription deregulation, and repetitive activation of STAT3 appeared responsible for the change. BRD4 accumulation prolongs transcription activity at targeted sites with a secondary stimulus by prolonging transcription factor binding and duration of transcriptions. This epigenetic memory may explain the greater risk of chronic inflammatory disease development in KSHV-infected individuals.

## Results

### Biological effects of prolonged vIL-6 exposure on gene transcription and phenotypes

**Fig 1:**
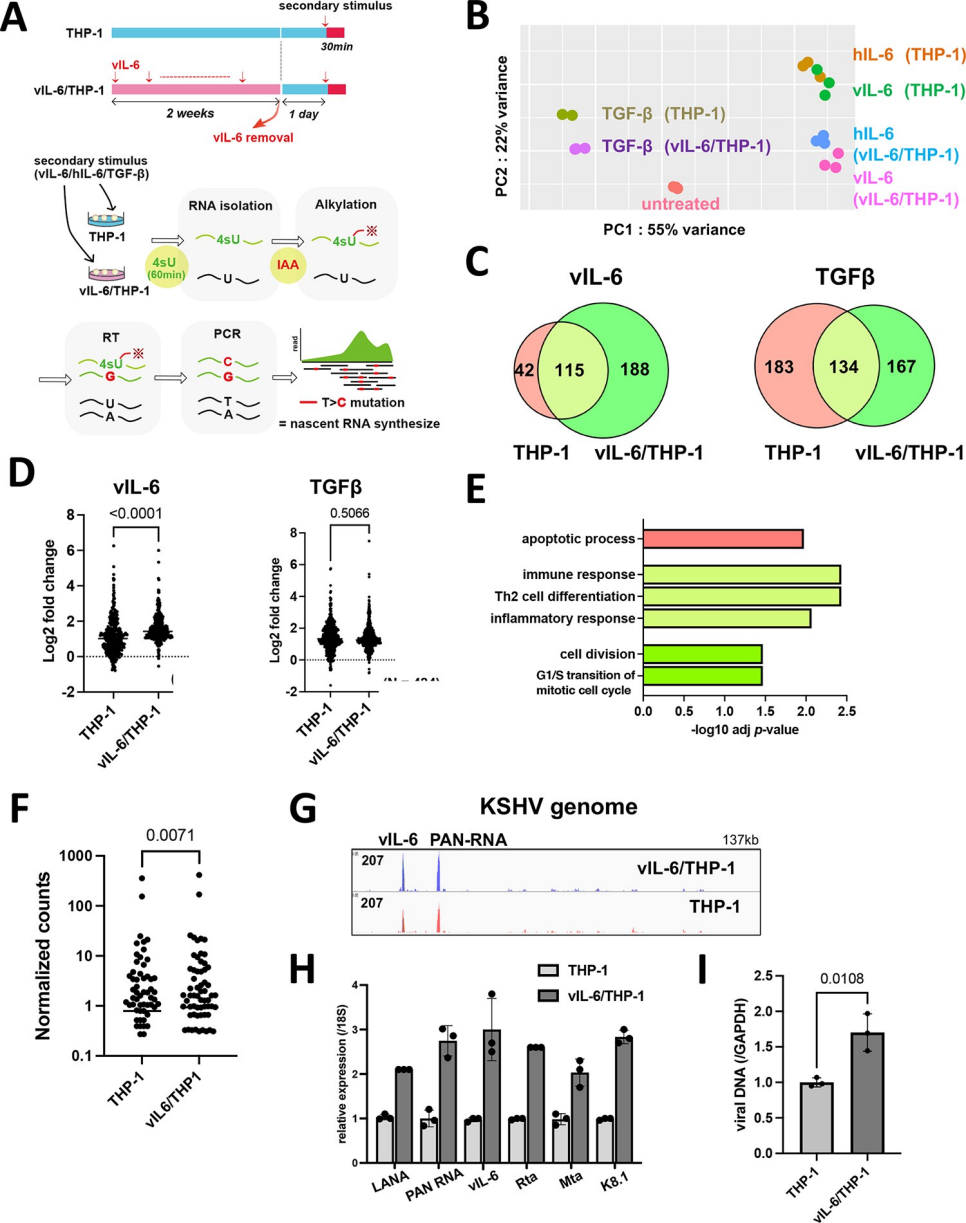
**Biological effects of prolonged vIL-6 exposure (A)** A schematic diagram of the THP-1 cell culture model. THP-1 cells were treated with or without vIL-6 every other day for 2 weeks (THP-1, vIL-6/THP-1). After withdrawal of the cytokine for 24 hours. vIL-6, hIL-6 and TGF-β were added for 30 minutes for secondary stimulation. Subsequently, 4-Thiouridine (4sU, 300 μM) was added to the culture media and the cells were incubated for 1 hr to label newly synthesized RNA. Newly synthesized RNA was then alkylated by iodoacetamide (IAA, 100mM). Total RNA was isolated for the following analyses. **(B)** Principal component analysis (PCA). Nascent transcribed gene expression in THP-1 and vIL-6/THP-1 cells was shown. vIL-6, hIL-6 and TGF-β were used as secondary stimulation. Untreated THP-1 cells were used as a control. The samples were represented by three biological replicates. The x and y axes show the percentage of variance explained by PC1 (55% variance) and PC2 (22% variance). **(C)** The number of upregulated genes (log2 fold change >1, adj *p*-value < 0.01) after vIL-6 stimulation (left) and TGF-β stimulation (right). Red and green circles represent THP-1 and vIL-6/THP-1 cells, respectively. **(D)** Comparison of up-regulated gene expression between parent THP-1 and vIL-6/THP-1 cells after stimulation with vIL-6 (left, N = 344) or TGFβ (right, N = 484). Data were analyzed using Wilcoxon matched-pairs signed ranked test and shown as median. **(E)** KEGG pathway analysis was performed on up-regulated genes (log2 fold change >1, adj *p*-value < 0.01) in either THP-1 or vIL-6/THP-1 cells with vIL-6 stimulation. Each bar represents the pathways that were enriched only in parental THP-1 cells (red), only in vIL-6/THP-1 cells (green), or commonly enriched (light green). Results are presented in descending order of the analysis. **(F)** Comparison of all KSHV gene expression between parent THP-1 and vIL-6/THP-1 cells after r219.KSHV infection (N = 88) for 72 hours. Data were analyzed using Wilcoxon matched-pairs signed ranked test and shown as median. **(G)** RNA-sequence reads aligned to the KSHV genome (NC 009333.1) in parent THP-1 (red) and vIL-6/THP-1 cells (blue) after r219.KSHV infection. **(H)** KSHV gene expression in parent THP-1 and vIL-6/THP-1 cells after r219.KSHV infection. THP-1 and vIL-6/THP-1 cells were infected with r219.KSHV for 72 hours. RNA was then collected and transcribed into cDNA for RT-qPCR. 18S rRNA expression was used for internal control. Data was analyzed using two-sided unpaired Student’s *t* test and shown as mean ± SD. **(I)** KSHV DNA copies 72 hours at r219.KSHV post-infection. GAPDH was used for internal control. Data was analyzed using two-sided unpaired Student’s *t* test and shown as mean ± SD.

In contrast to temporally regulated hIL-6 expression during wound healing, persistent KSHV infection leads to continuous vIL-6 production from infected cells. Especially, vIL-6 is detected in the serum of KICS patients and the hematopoietic cells in Castleman’s disease patients with a high KSHV viral load [22]. To study the biological consequences of vIL-6 exposure, we first purified recombinant vIL-6 protein from recombinant baculovirus-infected cells and confirmed its biological activity by STAT1 and STAT3 activation [23]. Previous reports showed that vIL-6 concentration in KICS patients was approximately 3ng/ml-30ng/ml [24]. Here, we used 100 ng/ml as a final concentration of vIL-6 to ensure the activation of downstream signaling. With STAT3 phosphorylation as a readout, 100 ng/ml is approximately ten times the minimum amount required for inducing STAT3 phosphorylation in 293 cells. With the recombinant vIL-6, we prepared a cell culture model with THP-1 cells, which were exposed to vIL-6 continuously for two weeks (we refer to vIL-6/THP-1 hereafter) **(Fig 1A)**. THP-1 cells, a representative monocyte cell line was employed because monocytes play a significant role in inflammatory responses [25]. Using the cell culture model, we compared the overall transcriptional profiles between vIL-6/THP-1 and parental THP-1 cells, followed by secondary stimulation with selected cytokines. We used vIL-6 and hIL-6, which can activate common downstream signaling pathways, and TGF, which has a non-overlapping pathway, as a secondary stimulation.

To evaluate the newly synthesized RNAs by cytokines stimulation, thiol (SH)-Linked Alkylation for the Metabolic sequencing of RNA (SLAM-seq) was employed [26]. vIL-6/THP-1 and parental THP-1 cells were unstimulated or stimulated by vIL-6, hIL-6 or TGF-β for 30 minutes, followed by incubation with 4-thiouracil (4sU) for 60 minutes to label transcribing RNAs during the incubation periods. In this experimental setting, sequence reads with mutations T>C identify the only nascent synthesized mRNA because 4sU incorporation with chemical alkylation forces incorporation of G instead of A at 4sU positions during the reverse transcription step [26]. Isolated sequence reads with T>C mutation(s) were then compared among control (non-stimulation), vIL-6, hIL-6, and TGF-β **(Fig 1A)**. As shown in **Fig 1B**, principal component analysis (PCA) demonstrated distinct cellular responses in vIL-6/THP-1 and also among stimuli. In response to vIL-6 stimulation, we observed that the number of upregulated genes (> 2-fold change, adjusted *p*-value < 0.01) in vIL-6/THP-1 was about twice as many as those with parental THP-1 cells (**Fig 1C, left**). Similar responses were also seen with hIL-6 as a secondary stimulation (**S1A Fig**). The vIL-6 and hIL-6 secondary stimulation showed very similar transcriptional profiles (**S1B Fig**) and downstream pathways (**S1C Fig**), suggesting that vIL-6 is clearly a functional homolog of hIL-6 as it was suggested before [27,28]. On the other hand, the number of upregulated genes with the TGF-β was slightly decreased in vIL-6/THP-1 compared to parental THP-1 cells (**Fig 1C, right**). In addition, the degree of upregulated gene expression by vIL-6 or hIL-6 was also increased to a greater extent in vIL-6/THP1 cells compared to parental THP-1 cells (**Figs 1D and S1D**), while there were no significant differences with TGF-β (**Fig 1D**). The results suggested that the enhanced transcription response is a pathway dependent. Gene Ontology (GO) analysis indicated that genes related to immune response, Th2 cell differentiation, and inflammatory response were commonly enriched by secondary vIL-6 stimulation in parental and vIL-6/THP-1 cells (**Fig 1E: light green**), while cell cycle and cell division pathway were additionally activated in vIL-6/THP-1 cells (**Fig 1E: green**). Consistent with the GO analysis, cell proliferation was increased in vIL-6/THP-1 cells measured by MTT assay (**S1E Fig**). Despite an increased number of responding genes with the prolonged vIL-6 exposure, the degree of STAT3 phosphorylation and gp130 expression (cell surface receptor for vIL-6) was not changed (**S1F and S1G Fig**).

Since KICS patients are characterized not only by increased high vIL-6 or hIL-6 but also by increased viral loads [6], we examined the effect on KSHV gene expression during de novo infection in vIL-6/THP-1 cells. For this, we infected THP-1 and vIL-6/THP-1 cells with r219.KSHV and performed total RNA-sequencing (RNA-seq) at 72 hours post-infection. The efficiency of KSHV infection to THP1 and vIL-6/THP-1 cells was confirmed to be approximately 40% by flow cytometry (**S1H Fig**), and there were no differences with vIL-6 incubation in infectivity. The result suggested that vIL-6 treatment did not sensitize THP-1 cells to KSHV infection. RNA-sequencing showed that overall KSHV gene expression was higher in vIL-6/THP-1 cells compared to parental THP-1 cells (**Fig 1F**). Moreover, among induced KSHV transcripts, vIL-6 and PAN RNA expression were significantly higher in vIL-6/THP-1 compared to parental THP-1 cells (**Fig 1G**). Interestingly, vIL-6 was transcribed at a similar amount with PAN RNA during de novo infection in THP-1 cells (**Fig 1G**). The results suggested that prolonged vIL-6 may support KSHV gene transcription, and the results were further confirmed by RT-qPCR in selected genes (**Fig 1H**). Viral DNA synthesis was also increased in vIL-6/THP-1 cells at 72 hours post infection (**Fig 1I**). The amount of infectious virion production after *de novo* infection was also examined. The THP-1 culture supernatant was transferred to fresh iSLK cells, and GFP-positive cell numbers were measured. However, no GFP-positive cells were observed from either of the culture supernatants, suggesting that KSHV primarily undergoes latent infection in THP-1 cells. Taken together, prolonged vIL-6 exposure enhances transcriptional response to secondary vIL-6 and hIL-6 stimulation, and also establishes a cell environment that favors KSHV gene transcription and DNA replication.

### Prolonged vIL-6 stimulation increased BRD4 occupancies on chromatin

**Fig 2:**
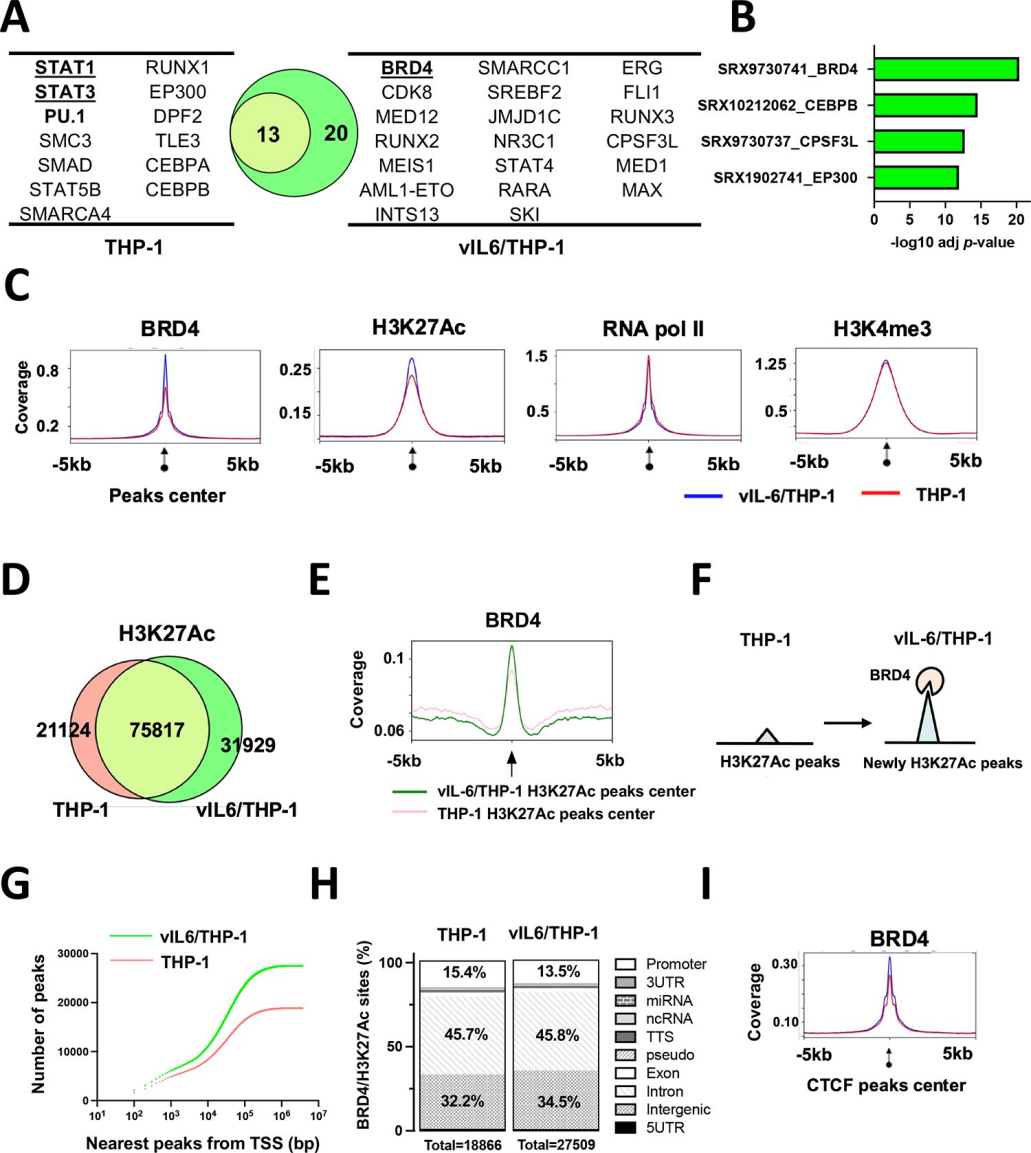
**Newly BRD4 recruitment and H3K27Ac translocation in vIl-6/THP-1 cells (A)** Enrichment analysis by CHIP-Atlas. Enrichment analysis was performed on the list of genes whose expression were up-regulated by vIL-6 stimulation. The parameters used were as follows; cell type class: blood, threshold for significance: 100, threshold for log10 adjusted *p* value < -10. The Venn diagram shows the relatedness and number of genes up-regulated after vIL-6 stimulation in parental THP-1 (red circle) and vIL-6/THP-1 cells (green circle). **(B)** The predicted transcription factors or mediators selectively enriched only in vIL-6/THP-1 cells. The results were limited to THP-1 cells in CHIP-Atlas database. **(C)** CUT&RUN signals in ±5-kbp windows around the center of peaks in vIL-6/THP-1 cells. The list of peaks (p-value < 10−^4^) was extracted using findPeaks (HOMER with default parameters). Enrichment is shown in vIL-6/THP-1 (blue line) and in THP-1 (red line) cells. Images were drawn by plotProfile (HOMER). **(D)** H3K27Ac marks in parental THP-1 and vIL-6/THP-1 cells. The number of H3K27Ac peaks in vIL-6/THP-1 and parental THP-1 cells are depicted by a Venn diagram. **(E)** BRD4 CUT&RUN signals in vIL-6/THP-1 cells. The green line indicates BRD4 peaks±5-kbp around the center of H3K27Ac peaks in vIL-6/THP1 cells while the pink line indicates those in parental THP-1 cells. **(F)** Schematic model of H3K27Ac translocation and BRD4 recruitment at newly emerged H3K27Ac regions in vIL-6/THP-1 cells. **(G)** Distances of the nearest TSS to overlapping peaks of BRD4 binding and H3K27Ac enrichment sites. Total peak counts are shown. *P* values were calculated by the Kolmogorov–Smirnov test. **(H)** Annotation of overlapping peaks between BRD4 and H3K27Ac enrichment sites. Each annotation and its proportion were calculated by annotatePeaks.pl (Homer; parameters; default). Promoter regions are defined as ± 1-kbp from TSS. **(I)** BRD4 CUT&RUN signals in ±5-kbp windows around the center of CTCF peaks in vIL-6/THP-1 cells (blue) and parental THP-1 cells (pink).

The studies above demonstrated that prolonged vIL-6 exposure increased the number of responding genes followed by vIL-6 or similar (hIL-6) stimuli without altering the amounts of signal-dependent transcription factor (STAT3). These results led us to examine chromatin modification changes induced by prolonged vIL-6 exposure. We first used CHIP-Atlas, a data mining tool for predicting proteins bound to promoters of upregulated genes based on a comprehensive epigenetic database, and we narrowed down possible epigenetic factors. As shown in **Fig 2A**, STAT1 and STAT3 were commonly identified as putative responsible transcriptional factors for the upregulation of promoter activity with vIL-6 stimuli, demonstrating the validity of this enrichment analysis. Transcription coactivators and several transcription factors, such as Cyclin dependent kinase 8 (CDK8), Mediator complex subunit 12 (MED12), and Bromodomain-containing protein 4 (BRD4), were enriched only in the genomic loci in vIL-6/THP-1 cells (**Fig 2A**). BRD4 is a member of the BET (bromodomain and extra terminal domain) family and an important coactivator that binds to H3K27Ac to mediate transcription regulation together with CDK8, MED12, and RNA polymerase II (RNAPII) [29]. Because BRD4 was identified as the most enriched transcription regulator with prolonged vIL-6 exposure (**Fig 2B**), we next performed Cleavage Under Targets & Release Using Nuclease (CUT&RUN) for BRD4 and for RNAPII to interrogate promoter activity. In addition, two active histone modification marks (H3K27Ac, H3K4me3) and CTCF were examined to reveal changes in chromatin modification landscape [30]. Parental THP-1 and vIL-6/THP-1 cells were compared in duplicate samples for statistical analyses. Consistent with the CHIP-Atlas results, BRD4 was enriched in the transcription start sites of up-regulated genes in vIL-6/THP-1 cells (**S2A Fig**). We also found that BRD4 and H3K27Ac peaks were increased across the genome in vIL-6/THP-1 cells, while RNAPII and H3K4me3 were unchanged (**Fig 2C**). Despite increasing BRD4 occupancies in chromatin, immunoblotting showed that the total amount of BRD4 protein, as well as H3K27Ac or H3K4me3 modifications remained comparable with prolonged vIL-6 exposure **(S2B Fig)**. The results suggested that BRD4 was recruited on the genome more in vIL-6/THP-1 cells. HOMER motif analysis for newly accumulated BRD4 genomic regions identified key monocyte transcription factors such as Elf4, ETS Transcription Factor 4, and PU.1(**S2C Fig**). PU.1 was also identified as a transcriptional factor likely to be involved in gene activation seen in prolonged vIL-6 exposure by SLAM-seq **(Fig 2A)**.

We next examined the position and association of the newly accumulated BRD4 with H3K27Ac to reveal the changes in active genomic domains. The CUT&RUN studies identified 31,929 newly established H3K27Ac peaks in vIL-6/THP-1 cells, while 21,124 peaks were no longer seen in the cells **(Fig 2D).** As shown in **Fig 2E**, BRD4 was enriched more and sharply at the centers of H3K27Ac peaks in vIL-6/THP-1 cells. The genomic regions, where BRD4 and H3K27Ac peaks overlap, were also increased from 18,886 to 27,509 by prolonged vIL-6 exposure (**S2D Fig**), suggesting that newly accumulated BRD4 was recruited in newly established H3K27Ac loci in vIL-6/THP-1 cells (**Fig 2F**). RNAPII and H3K4me3 were also enriched in the genomic regions, where BRD4 and H3K27Ac peaks overlapped(**S2E Fig**). Closer examination of these enriched sites demonstrated that while the overall number of BRD4/H3K27Ac overlapped peaks around TSS was increased (**Fig 2G**), the relative abundance of the peaks at the promoter region decreased from 15.4% to 13.5% in vIL-6/THP-1 cells (**Fig 2H**). These results may suggest that a larger fraction of newly generated active loci are located in intragenic regions. The CUT&RUN results with the CTCF antibody also suggested that prolonged vIL-6 stimulation increased overall CTCF co-occupancies with BRD4 across the genome (**Fig 2I**), suggesting the increased numbers of activated chromatin hubs in vIL-6/THP-1 cells. The GO analyses suggested that the newly established BRD4 and H3K27Ac peaks were localized to promoter regions of genes associated with DNA damage stimulus and cell division, such as cyclin E1; the results were also consistent with the SLAM-seq analysis **(S2F and S2G Fig)**.

### Enhanced response to LPS via prolonged vIL-6 stimulation

**Fig 3:**
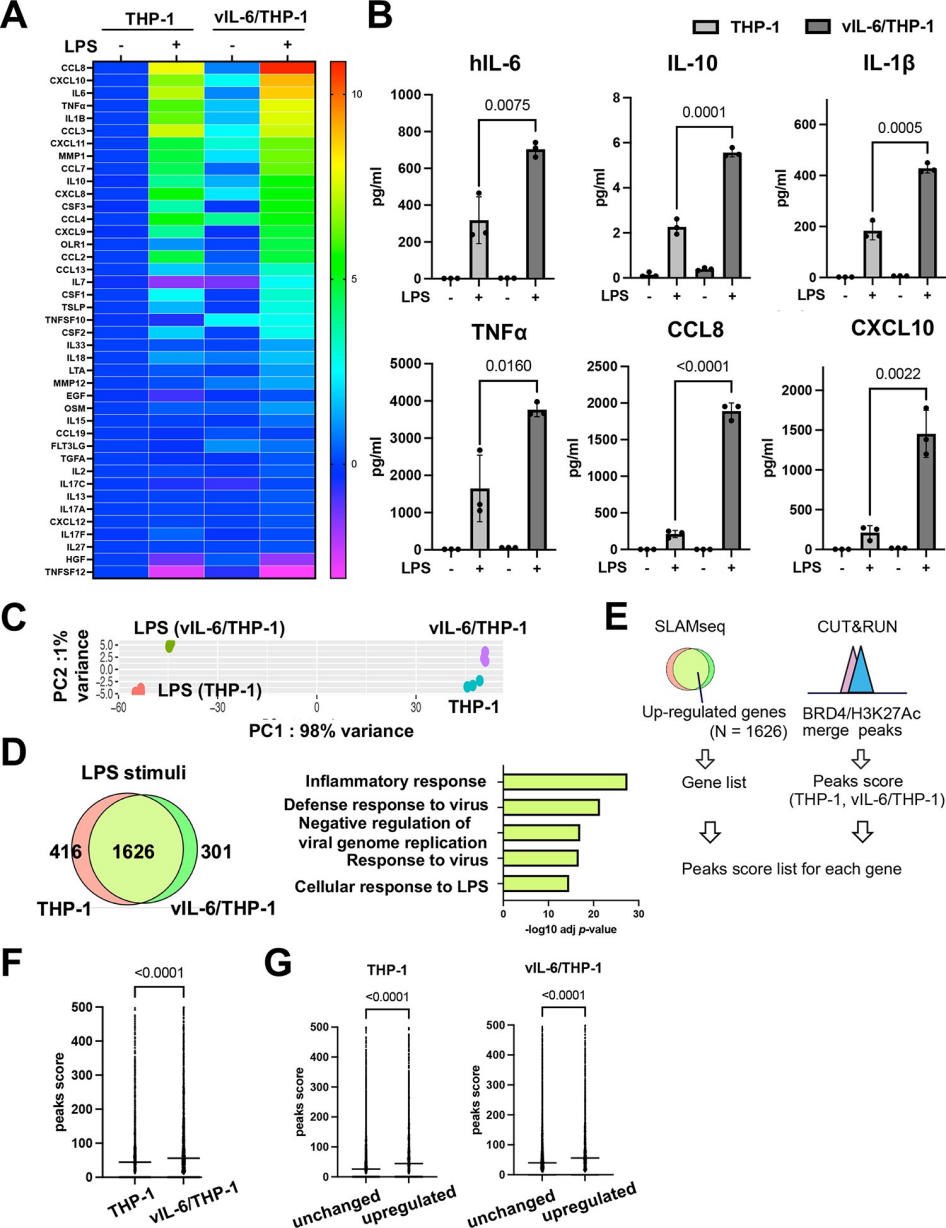
**Prolonged vIL-6 exposure enhances inflammatory response to LPS through the novel accumulation of BRD4 and H3K27Ac (A)** Heatmap showing the results of Olink Target 48 Cytokine panel analysis. LPS (100ng/ml) was added to parental THP-1 or vIL-6/THP-1 cells for 6 hours. Cytokine production in untreated THP-1 cells was set as 1 and log_2_ fold activation relative to untreated THP-1 cells is shown. Samples were prepared in triplicate, and the mean values were shown. **(B)** Individual inflammatory cytokine production determined by Olink proximity extension assay. Data was analyzed using two-sided unpaired Student’s *t* test and shown as mean ± SD. **(C)** PCA based on nascent transcribed gene expression in THP-1 cells and vIL-6/THP-1 cells after LPS stimulation. The samples were represented by 3 biological replicates. The x and y axes show the percentage of variance explained by PC1 (98% variance) and PC2 (1% variance). **(D)** The number of up-regulated genes (log2 fold change >1, adj *p*-value < 0.01) in THP-1 and vIL-6/THP-1 cells after LPS stimulation (left) and KEGG pathway analysis performed on commonly up-regulated genes (right). **(E)** Schematic diagram for comparing peaks score of up-regulated genes between THP-1 and vIL-6/THP-1 cells. Commonly up-regulated genes by LPS stimulation were obtained from SLAMseq data and BRD4/H3K27Ac merge peaks score were obtained from CUT&RUN data. The peak scores were associated with each up-regulated gene. **(F)** BRD4/H3K27Ac merge peak score for commonly upregulated genes in THP-1 and vIL-6/THP-1 cells (N = 1626). Data were analyzed using Wilcoxon matched-pairs signed ranked test and shown as median. **(G)** BRD4/H3K27Ac peak score between up-regulated genes (N = 1626) and unchanged genes (log2 fold change >1 and < -1, adj *p*-value > 0.01) (N = 4582) in THP-1 (left) and vIL-6/THP-1 cells (right). Data were analyzed using the Mann-Whitney test and shown as the median.

Synergistic interactions between NF-κB and STAT3 induce the hyperactivation of NF-κB followed by the production of various inflammatory cytokines such as hIL-6 [16]. KSHV-infected cells are known to have elevated NF-κB activation, and STAT pathways are constitutively activated in primary effusion lymphomas [31,32]. Accordingly, we examined if repetitive STAT activation by vIL-6 exposure alters NF-κB mediated cell responses. To study this, we stimulated vIL-6/THP-1 cells with LPS, a strong inducer of NF-κB activation. We first confirmed that 100 ng/ml of LPS stimuli for 6 hours is sufficient for detecting hIL-6 production by ELISA assay **(S3A Fig)**, and selected 6 hours as a time point to collect samples that should minimize the secondary effects from the newly produced cytokines with LPS stimuli. We measured a series of downstream inflammatory cytokines produced with Olink, a multiplex proximity extension assay (PEA) [33]. The results showed that LPS upregulated the expression of 21/41 inflammatory cytokines (log2 fold change >1) in parental THP-1 cells (**Fig 3A**). Among them, the expression of 15 cytokines, such as hIL-6, IL-10, IL-1β, and CCL8, was enhanced in vIL-6/THP-1 cells compared to parental THP-1 cells (**Fig 3A and 3B**). Interferon-α stimulation also induced more cytokines in vIL-6/THP-1 cells similar to that of LPS stimulation (**S3B and S3C Fig**), suggesting that prolonged vIL-6 exposure to monocytes sensitized cells to secondary innate immune stimuli.

**Fig 4:**
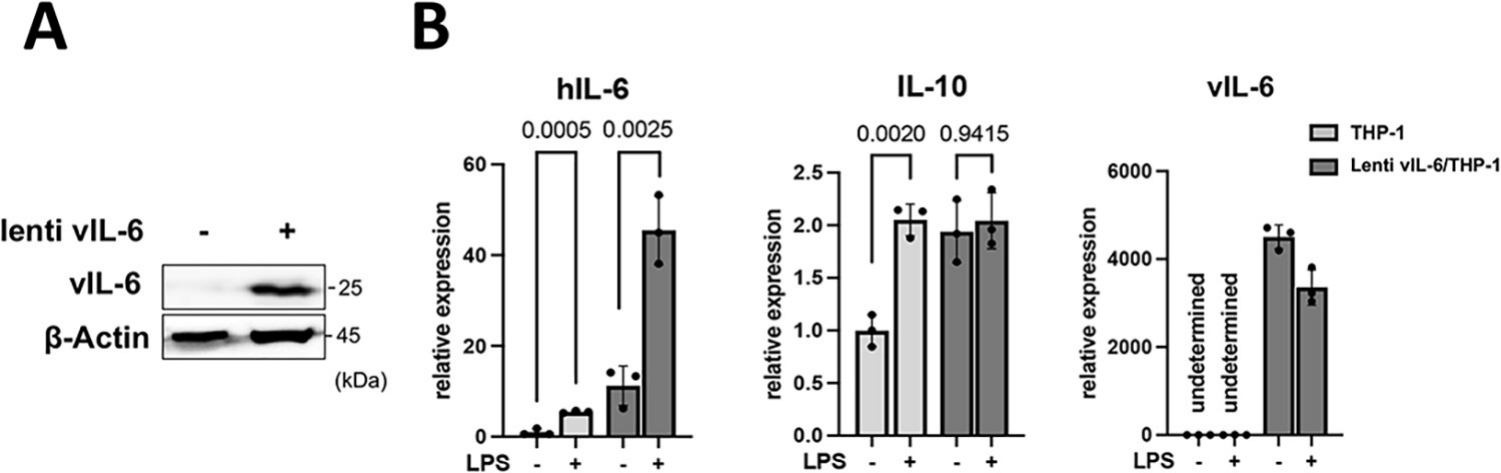
Activation of inflammatory gene expression by vIL-6 expression in cells. **(A)** Immunoblotting of THP-1 cells transduced with plenti4/V5-DEST expressing vIL-6. **(B)** hIL-6, IL-10 and vIL-6 genes expression in lenti vIL-6/THP-1 and parental THP-1 cells. Cells were stimulated with LPS for 1 hour and total RNA was harvested. Fold activation over parental THP-1 without stimulation is shown. lenti vIL-6 THP-1: vIL-6 expressing lentivirus transduced THP-1 cells, THP-1: parental cells.

The transcription profile in response to LPS was also examined by SLAM-seq analysis. LPS and 4sU were added to parental THP-1 cells or vIL-6/THP-1 cells simultaneously and incubated for 6 hours to label nascent transcribed RNAs. PCA demonstrated that LPS strongly changed the gene transcription profile, and the variance between THP-1 and vIL-6/THP-1 cells was further increased by LPS stimulation (**Fig 3C**). As expected, the inflammatory response was identified as the most enriched pathway in commonly up-regulated genes (N = 1626) (**Fig 3D**). Next, we investigated whether the increased BRD4 and H3K27Ac occupancies in vIL-6/THP-1 cells contributed to the increased inflammatory gene transcription by LPS stimulation. Commonly up-regulated genes were extracted, and the peak scores of the BRD4 and H3K27Ac overlapping regions in THP-1 and vIL-6/THP-1 cells were compared (**Fig 3E**). As shown in **Fig 3F**, co-occupancy of BRD4 and H3K27Ac was indeed associated with increased transcription activation with LPS. The increased responses to LPS stimuli were also associated with an increased BRD4 and H3K27Ac co-occupancy compared to genes whose expression was not changed (**Fig 3G**). Finally, we also examined the effect of de novo vIL-6 expression. This is because previous studies showed that vIL-6 can function in infected cells without secreting in serum. The vIL-6 could accumulate in ER and be regulated primarily in KSHV-infected cells [34,35]. We first established the THP-1 cells that stably express vIL-6 by lentivirus infection **(Fig 4A**), and tested the inflammatory response after LPS stimulation. We found that de novo vIL-6 expression also increased the hIL-6 expression, similar to repetitive exogenous vIL-6 exposure **(Fig 4B**). The production of hIL-6 is remarkably higher compared to parental THP-1 cells, and this difference becomes even more pronounced upon LPS stimulation **(Fig 4B**). IL-10 expression seems saturated before LPS stimulation in THP-1 cells with vIL-6 expression **(Fig 4B**). This suggests that the continuous activation of the STAT pathway by prolonged exposure to vIL-6, regardless of paracrine or autocrine fashion, increases inflammatory responses with secondary inflammatory stimuli.

### BRD4 is responsible for prolonging the hIL-6 transcription burst with LPS

**Fig 5:**
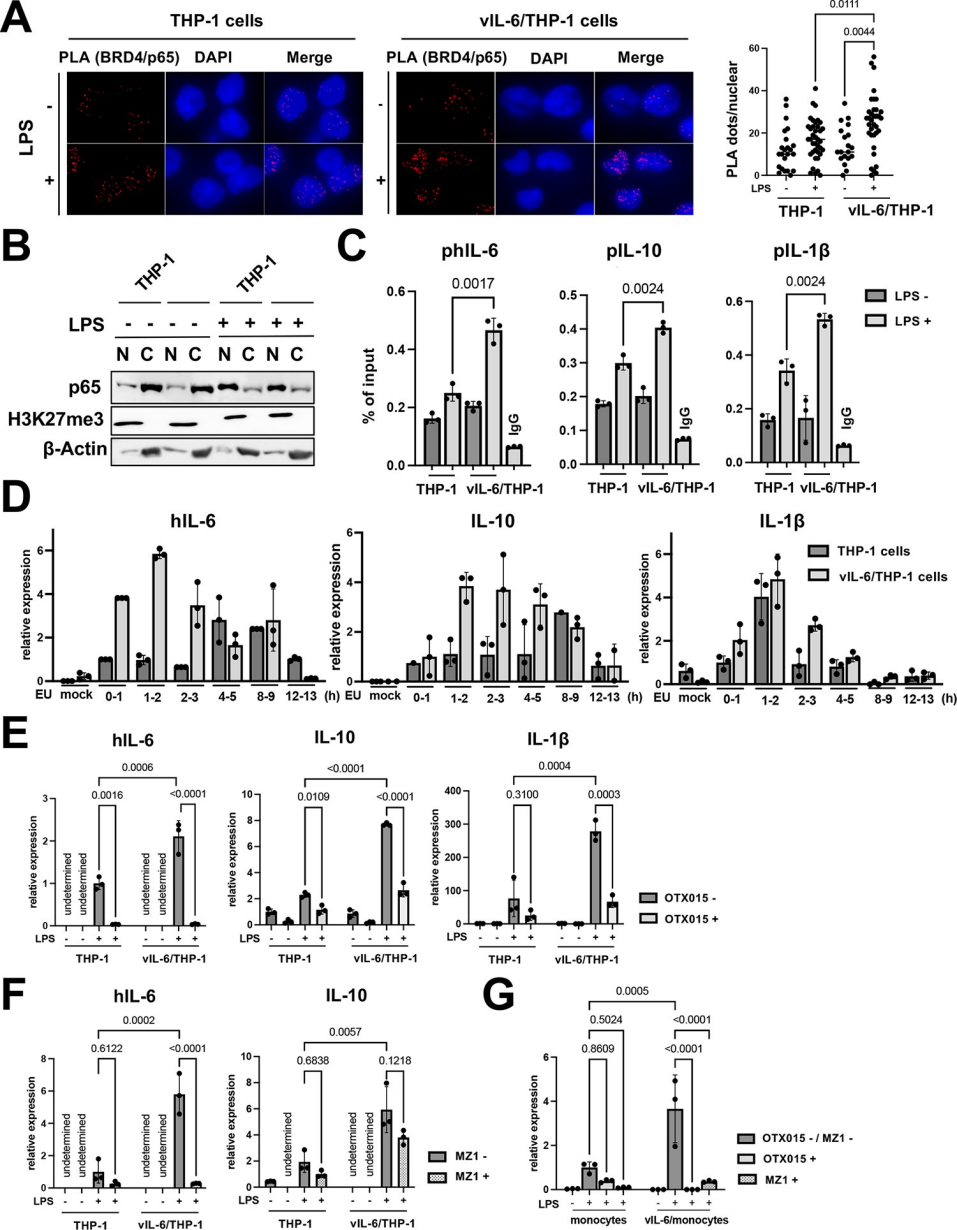
**Association of BRD4 and p65 is responsible for prolonging hIL-6 transcription burst by LPS (A)** Proximity extension assay (PLA) to visualize BRD4 and p65 interaction. THP-1 and vIL-6/THP-1 cells were incubated with LPS (100ng/ml) for 6 hours, fixed by 4% PFA/PBS for 15min, permeabilized by 0.15% TritonX/PBS for 10min and PLA was performed. Representative images of PLA (red dots) and quantification of PLA are shown (right). Data were analyzed using a one-way ANOVA test. **(B)** Immunoblotting of p65, H3K27me3, and β-actin in the nuclear and cytoplasmic fractions. THP-1 and vIL-6/THP-1 cells were incubated with LPS (100ng/ml) for 6 hours and cell fractionation was performed. **(C)** The levels of p65 enrichment in the hIL-6, hIL-10, and IL-1β promoter regions were detected using ChIP-qPCR. Normal Rabbit IgG antibody was used as a negative control. Data was analyzed using two-sided unpaired Student’s *t* test and shown as mean ± SD. **(D)** Nascent RNA expression in THP-1 and vIL-6/THP-1 cells after LPS stimulation. Cells were incubated with LPS and ethynyl uridine (EU) was added at 0h, 1h 2h, 4h, 8h, and 12h after LPS stimulation for 1 hour. Mock samples were prepared without EU for 1 hour incubation with LPS. **(E)** The hIL-6, IL-10, and IL-1β gene expression level in response to OTX-015 treatment. THP-1 and vIL-6/THP-1 cells were incubated with OTX-015 (40nM) for 4 hours followed by LPS (100ng/ml) for 1 hour. RNA was then collected and transcribed into cDNA for RT-qPCR. 18S rRNA expression was used for internal control. Data were analyzed using a one-way ANOVA test. **(F)** The hIL-6 and IL-10 gene expression level in response to MZ1 (250nM) treatment. Sample preparation and data processing were the same as OTX-015 treatment. **(G)** hIL-6 gene expression level in response to OTX-015 and MZ1 treatment. Primary monocytes were exposed to vIL-6 (100ng/ml) every other day for 1 week (vIL-6/monocytes). Cells were then incubated with OTX-015 (40nM) or MZ1 (250nM) for 4 hours followed by LPS (100ng/ml) for 1 hour. RNA was then collected and transcribed into cDNA for RT-qPCR. 18S rRNA expression was used for internal control. Data were analyzed using a one-way ANOVA test.

**Fig 6:**
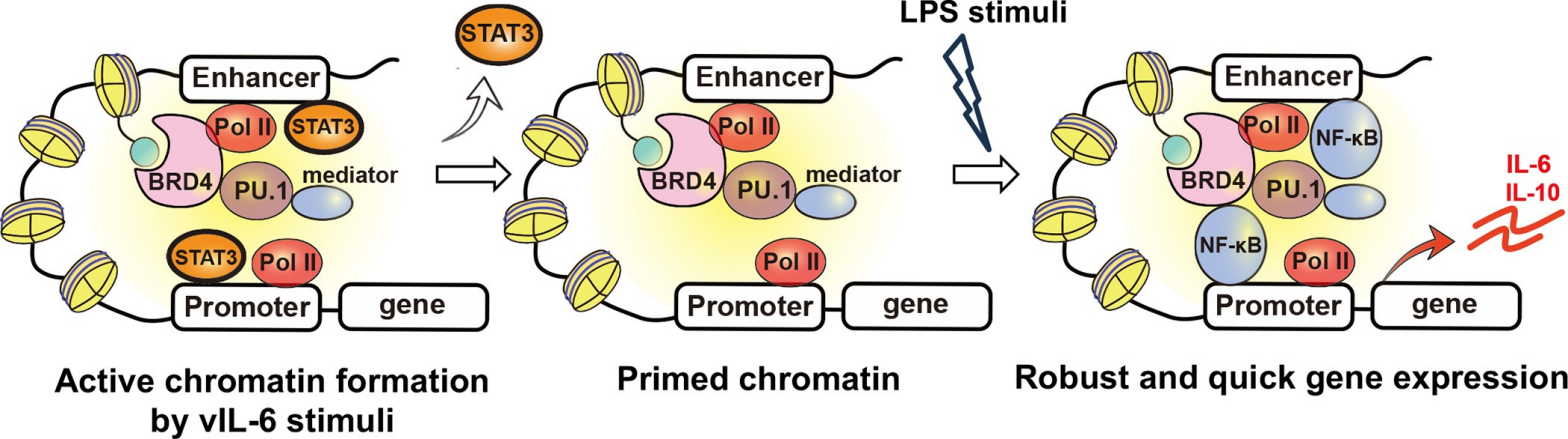
**Schematic model of transcription reprogramming by prolonged vIL-6 exposure** Signal-dependent transcription factors such as STAT3 are recruited at up-regulated gene promoters by vIL-6, and BRD4 and H3K27Ac are increasingly co-occupied at those promoters by prolonged vIL-6 exposure. After removing the stimulus, STAT3 is removed from the promoters; however, the activated H3K27Ac modification and BRD4 remained and established a "primed" chromatin state for subsequent activation. Subsequent LPS stimulation activated hIL-6 or IL-10 genes that had been primed by vIL-6, more quickly and robustly.

Because BRD4 was enriched in the promoter regions of hIL-6, hIL-10 and IL-1β with the prolonged vIL-6 exposure **(S4A Fig)** and was associated with increased production of cytokines in culture media **(Fig 3A)**, we next studied how BRD4 recruitment contributes to increasing transcripts. We speculated that increased occupancies of BRD4, which contains an intrinsically disordered domain to form liquid-liquid phase separation (LLPS) [36], may increase the recruitment and duration of p65 binding at promoter regions. This mechanism may extend the duration or robustness of transcription after stimulation. Accordingly, we examined the recruitment of p65 after LPS stimuli in vIL6/THP-1 cells and compared it with parental THP-1 cells. The proximity ligation assay showed that the association between BRD4 and p65 was increased after LPS stimulation in vIL6/THP-1 cells (**Fig 5A**), while the total p65 amount in the nucleus after LPS stimulation was not significantly changed in vIL-6/THP-1 cells (**Fig 5B**). ChIP-qPCR also showed increased p65 recruitment on the hIL-6, IL10, and IL-1β promoters by LPS stimuli in vIL6/THP-cells (**Fig 5C**). Transcription frequencies were also determined by qRT-PCR analysis of nascent RNA **(S4B Fig)**. As shown in **Fig 5D**, hIL-6, IL-10, and IL-1β transcripts were produced more rapidly (hIL-6 and IL-10) and/or continuously (IL-1β) in vIL-6/THP-1 cells after stimulation. Importantly, BRD4 occupancies are responsible for the increased transcription, because OTX-015, a BRD4 inhibitor, drastically suppressed the transcription in both parental THP-1 and vIL-6/THP-1 cells. The effect was more pronounced in vIL-6/THP-1 cells (**Fig 5E**). To further confirm the significance of BRD4, we used another BRD4 selective inhibitor, MZ1. MZ1 was reported to be a more specific inhibitor for the BRD4 [37,38] (**S4C Fig**). As shown in **Fig 5F**, MZ1 also suppressed hIL-6 transcription. Although inhibition of hIL-10 transcription was also seen, the extent of inhibition was not as much as hIL-6, which did not meet statistical significance **(Fig 5F)**. Finally, we confirmed similar biological effects in human monocytes to exclude cell line bias. As similar to THP-1 cell model, hIL-6 transcription in response to LPS was enhanced with prolonged vIL-6 exposure, and the responses were again suppressed by the BRD4 inhibitors **(Fig 5G)**. Taken together, prolonged vIL-6 exposure remodels the chromatin to favor the response to inflammatory secondary stimuli, and the establishment of active chromatin hubs with BRD4 recruitment is critical for the reactivation (**Fig 6**).

## Discussion

In this study, we applied SLAM-seq for most of the RNA-seq experiments to increase the resolution to identify direct target genes. A short window after the respective stimuli was utilized to examine robustness and differences made in direct target gene expression with prolonged vIL-6 stimuli. The study clearly demonstrated that direct target genes such as *BCL3*, *BCL6*, *and CISH*, are shared with hIL-6 (**S1B Fig**). These genes are known as STAT3 target genes [39–41], validating our SLAM seq analysis. KEGG pathway analysis also identified the common pathways including inflammatory response and apoptotic process by vIL-6 and hIL-6 stimulation. The results suggest that these two signaling ligands are interchangeable, making it easier to have continuously stimulated IL-6 signaling in KSHV infected individuals.

Cytokines that activate STAT3 (e.g. hIL-6) and NF-κB (e.g. TNF-α) synergistically increase the production of various inflammatory cytokines, called IL-6 Amp. Since vIL-6 has very similar biological activity as hIL-6, vIL-6 may bypass hIL-6 expression to trigger the IL-6 Amp in vivo. The vIL-6 can bind to gp130 without high affinity IL-6 receptor [42,43]. This contrasts hIL-6, which must bind to its high-affinity receptor before a signaling complex can be formed with its signal transducer, gp130 [1,44]. Importantly, the gp130 is widely expressed in human tissues. The notion that vIL-6 activates the downstream JAK/STAT pathway without requiring binding to the classical gp80 receptor [45–48] suggests that vIL-6 would initiate signaling in a wider variety of cell types [47,49]. In addition, previous studies by Dr. Nicholas’s group further demonstrated that vIL-6 can function intracellularly via activation of gp130 at the endoplasmic reticulum in infected cells [34,35]. The study suggested that vIL-6 may support the growth and survival of KSHV-infected cells in an autocrine manner, and such unique signaling activities contribute to the maintenance of latently infected cells without alarming bystander cells. In this regard, lentivirus transduction of vIL-6 similarly enhances inflammatory responses to secondary stimuli (**Fig 4).** Our recent study with KSHV primary monocyte infection model agreed well with this THP-1 vIL-6 culture model, in which prolonged vIL-6 stimuli shifted the cell transcription program towards cell growth and anti-apoptotic signaling (**Figs 1E and S1E**). More importantly, the phenotypes were only seen in wild-type KSHV primary infection but not in vIL-6 stop KSHV infected monocytes [23].

The immune activation by ligands is often transient, which is deactivated by cell receptor internalization and/or dephosphorylation of transcription factors [50,51]. Secondary activation, immediately following initial activation, often results in decreased cell responses. However, continuous stimulation cycle appears to leave transcription-related enzymes on the chromatin at the activated promoters, resulting in rapid and prolonged transcription, hence increased cytokine productions. Accordingly, there may be two types of vIL-6-mediated STAT3 signaling regulations: transient/local gene activation and persistent/global remodeling. Persistent KSHV infection with vIL-6 expression is likely to associate more with the latter, which may be a reason for the induction of dysfunctional macrophage by wild-type KSHV infection (but not with vIL-6Stop KSHV) [23]. Chronic inflammation is known to lead to a more robust inflammatory response, which is described as immunological training [52]. In immunological training, the primary stimulus primes specific regulatory elements in enhancer regions and establishes a chromatin landscape, which allows faster and increased transcriptional activation of the secondary stimulus. Immunological training therefore induces long-lasting epigenetic remodeling of regulatory elements [53,54]. This remodeling includes changes in activating histone marks such as H3K27Ac and H3K4me3 [53,54]. Accordingly, KSHV vIL-6 seems to utilize the immunological training mechanisms to establish a suitable nuclear environment for latent infections.

What would be the evolutional advantage for maintaining vIL-6 in KSHV genome? Interestingly, vIL-6 is reported to be constitutively expressed in some cell populations during latent infection [11,55]. We think that immunological training effects are utilized to establish active latent chromatin for inducible lytic gene expression. The BRD4, H3K27Ac, and H3K4me3 are all accumulated at the KSHV ori-Lyt region in latent chromatin [56]. It will be exciting to study if KSHV utilizes host defense mechanisms to recruit BRD4 on KSHV enhancer to establish a "primed" latent chromatin for future reactivation; it was partly seen in increased KSHV transcription in vIL-6/THP-1 cells (**Fig 1H and 1I**).

The mechanism of inflammation exacerbation in KS and the development of therapies to control the progression have attracted much attention in recent years [57–59]. Pomalidomide, an immunomodulatory drug, has been reported to be effective against KSHV-infected patients [60–62]. The pomalidomide suppresses the production of inflammatory cytokines such as IL-1β, TNF-α [63]. Although our current study focused on prolonged vIL-6 exposure with a monocyte model, prolonged exposure of host and viral inflammatory cytokines in other cell types may also have similar epigenetic effects that favor inflammatory and KSHV reactivations, establishing pathogenic feedforward loops. Disruption of the pathogenic feedforward loops by easing inflammatory responses may be therefore valuable for suppressing KSHV reactivation and treating the lesions; it is indeed seen in clinical trials [60–62].

In summary, we demonstrated that long-term vIL-6 exposure increased BRD4 and H3K27Ac co-occupancies at inflammatory-related genes, resulting in enhanced inflammatory responses to LPS. BRD4 is the center of the altered cell responses caused by prolonged vIL-6 exposure, in which the pre-occupied BRD4 enabled transcription factors to interact more rapidly and steadily on the promoters. Changes and therapeutics to rewind the pathogenic feedforward loops established by the inflammatory microenvironment might be a promising avenue for further KSHV research. Moreover, animal models with continuous vIL-6 exposure should reveal how continued inflammatory stimuli polarize the immune cell community and change the behavior of the individual cells. Although continued treatment with BET inhibitors induces significant transcription reprogramming and rapidly induces chemotherapeutic resistant cells [64,65], short-term treatment with well-scheduled drug holidays may be beneficial to reverse the immune trained memory initiated by KSHV vIL-6.

## Materials and methods

### Ethics statement

Peripheral blood mononuclear cells (PBMCs) were recovered from Leukoreduction Chambers from Apheresis Collections purchased from Vitalant Research Institute. Those Leukoreduction Chambers were to be discarded during the normal course of volunteer blood donations collected from healthy donor subjects using FDA-approved collection methods under an IRB-approved protocol at Vitalant Research Institute, San Francisco, CA, with written informed consent provided. Since these cells were not obtained for experimentation and the donors are anonymous, use of these cells was not considered human subject research requiring Institutional Review Board approval.

### Chemicals, reagents and antibodies

Dulbecco’s modified minimal essential medium (DMEM), RPMI 1640 medium, fetal bovine serum (FBS), phosphate-buffered saline (PBS), Trypsin-EDTA solution, and 100 X penicillin–streptomycin–L-glutamine solution were purchased from Thermo Fisher. Puromycin and G418 solution were obtained from InvivoGen. Hygromycin B solution was purchased from Enzo Life Science.

The following antibodies were used for CUT & RUN, immunoblotting and flow cytometry: rabbit anti-BRD4 (Cell Signaling Technology (CST), E2A7X), rabbit anti-RNAPII (Millipore Sigma, clone CTD4H8), rabbit anti-H3K27ac (CST, clone D5E4), rabbit anti-H3K4me3 (Cell Signaling, clone C42D8), mouse anti-β-actin (Santa Cruz 47778), rabbit anti-gp130 (CST), rabbit IgG (CST, clone DA1E). Rabbit anti-vIL-6 was a gift from Dr. Robert Yarchoan (NIH/NCI).

The following cytokines, human IL-6 (GenScript), TGF-β (Invitrogen), IFN-α (GenScript), and LPS (Sigma), were purchased and used. OTX-015 and MZ1 were purchased from Millipore Sigma and abcam, respectively. For lentivirus production, vIL-6 gene was amplified by PCR using wild type KSHV-BAC as a template, and cloned into pLenti4/V5-DEST vector (Invitrogen) at the SpeI and XhoI sites. Successful insertion was confirmed by DNA sequencing. In addition to vIL-6 expressing pLenti4/V5-DEST vector was cotransfected with packaging vectors, psPAX2 and pMD2.G, to 293FT cells for lentivirus production.

### Cells

THP-1 cell lines were obtained from ATCC. Human CD14+ monocytes were purchased from Millipore-Sigma. Cells were maintained in an RPMI 1640 medium supplemented with 1% FBS with 2 mM L-glutamine, 100 U/ml penicillin, and 100 μg/ml streptomycin at 37°C in a humidified 5% CO_2_. THP-1 cells were passaged two to three times per week when reaching 1 x 10^6^ cells/ml. For vIL-6/THP-1 cells and vIL-6/monocytes, cells were treated with vIL-6 100ng/ml every other day for 2 weeks or 1 week.

### Purification of recombinant protein

Purification of recombinant protein were performed with the BAC-to-Bac system as previously described [66,67]. Briefly, vIL-6 cDNA was synthesized, which included a Flag tag and cloned into pFAST-BAC vector. Recombinant baculovirus bacmid DNA was then transfected into *Spodoptera frugiperda* (Sf9) cells by using polyethylenimine (Sigma) and recombinant viruses were subsequently amplified once. Expression of recombinant proteins was confirmed by immunoblotting with anti-vIL-6 monoclonal antibody. Large-scale cultures of Sf9 cells (50 ml) were infected with recombinant baculovirus at a multiplicity of infection of 0.1–1.0, and cells were harvested 48 h after infection. Recombinant proteins were then purified after lysing infected Sf9 cell with high slat lysis buffer (Tris-HCl (pH7.5), 500 mM NaCl, 1% Triton X-100, 5% glycerol, and protease inhibitor cocktail). Cell lysates were cleared by centrifuge (7,000 rpm x 15 min at 4 °C) and incubated with Flag-agarose beads. (Thermo Fisher). The purity and amount of protein were measured by SDS-PAGE and coomassie blue staining using bovine serum albumin (BSA) as a standard.

### SLAM-seq

SLAM-seq [26] was performed using the SLAMseq Kinetics Kit (Lexogen GmbH, Vienna, Austria) according to the manufacturer’s standard protocol.

Briefly, biological replicate cultures of THP-1 and vIL-6/THP-1 cells were incubated with vIL-6 (100μg/ml) for 30 min. Subsequently, 4-Thiouridine (s4U; 300 μM) was added to the culture media, and the cells were incubated for 1 h to label newly synthesized RNA. As for the LPS (100ng/ml) stimulation, s4U was added to the culture media simultaneously with LPS, and cells were incubated for 6 hours. Total RNA was isolated, and then the 4-thiol groups in the s4Uracil-labeled transcripts were alkylated with iodoacetamide (IAA). QuantSeq 3′ mRNA-Seq (FWD) (Lexogen, Inc.) Illumina-compatible, indexed sequencing libraries were prepared from alkylated RNA samples (100 ng) according to the manufacturer’s protocol for oligo(dT)-primed first-strand cDNA synthesis, random-primed second-strand synthesis, and library amplification. Libraries were multiplex sequenced (1 × 100 bp, single read) on an Illumina HiSeq 4000 sequencing system. SLAM-Seq datasets were analyzed using the T > C conversion-aware SLAM-DUNK (Digital Unmasking of Nucleotide conversion-containing k-mers) pipeline utilizing the default parameters [26,68]. Briefly, nucleotide conversion-aware read mapping of adapter- and poly(A)-trimmed sequences to the human GRCh38/hg38 reference genome assembly was performed with NextGenMap [69]. Alignments were filtered for those with a minimum identity of 95% and minimum of 50% of the read bases mapped. For multi-mappers, ambiguous reads and non-3′ UTR alignments were discarded, while one read was randomly selected from multimappers aligned to the same 3′ UTR. SNP calling (coverage cut-off of 10X and variant fraction cut-off of 0.8) with VarScan2 [70] was performed to mask actual T > C SNPs. Non-SNP T > C conversion events were then counted and the fraction of labeled transcripts was determined. All results were used for downstream analyses, such as nascent transcript analysis and differential expression analysis (DESeq2) [71].

### Bioinformatics analysis of SLAM-seq data

The UCSC Genome Browser was used to convert RefSeq IDs to gene symbols (refGene). The resulting data were first filtered by log2 fold change and sorted by adjusted p-value from lowest to highest. The resulting up-regulated genes (log2 fold change >1 and adjusted *p*-value <0.01) were extracted. For functional enrichment analysis (KEGG pathway analysis), the official gene symbols were applied in DAVID web service tools [72]. For transcription factor analysis, gene symbols were submitted to ChIP-Atlas [73] to analyze common regulators and predict transcription factor binding. The following parameters were used; Organism (annotation): Homo sapiens (hg38), Experiment type; TFs and others, Cell type Class; Blood, Threshold; 100. The output factors were then listed and plotted in a Venn diagram.

### RNA-sequencing

vIL-6/THP-1 and parental THP-1 cells were infected with or without r219.KSHV for 72h and RNA was purified using the Quick-RNA miniprep kit (Zymo Research, Irvine, CA, USA). Indexed, stranded mRNA-seq libraries were prepared from total RNA (100 ng) using the KAPA Stranded mRNA-Seq kit (Roche) according to the manufacturer’s standard protocol. Libraries were pooled and multiplex sequenced on an Illumina NovaSeq 6000 system (150-bp, paired-end, >30 × 106 reads per sample). RNA-Seq data was analyzed using a Salmon-tximport-DESeq2 pipeline. Raw sequence reads (FASTQ format) were mapped to the reference human genome assembly (GRCh38/hg38, GENCODE release 36) and quantified with Salmon [74]. Gene-level counts were imported with tximport [75] and differential expression analysis was performed by DESeq2 [71], which are visualized by Venn diagram.

### Cleavage Under Targets and Release Using Nuclease (CUT&RUN)

CUT&RUN [30] was performed essentially by following the online protocol developed by Dr. Henikoff’s lab with a few modifications to fit our needs. Cells were washed with PBS and wash buffer [20 mM HEPES-KOH pH 7.5, 150 mM NaCl, 0.5 mM Spermidine (Sigma, S2626), and proteinase inhibitor (Roche)]. After removing the wash buffer, cells were captured on magnetic concanavalin A (ConA) beads (Polysciences, PA, USA) in the presence of CaCl2. Beads/cells complexes were washed three times with digitonin wash buffer (0.02% digitonin, 20 mM HEPES-KOH pH 7.5, 150 mM NaCl, 0.5 mM Spermidine and 1x proteinase inhibitor), aliquoted, and incubated with specific antibodies (1:50) in 250 μL volume at 4°C overnight. After incubation, the unbound antibody was removed with digitonin wash buffer three times. Beads were then incubated with recombinant Protein A/G–Micrococcal Nuclease (pAG-MNase), which was purified from *E*.*coli* in 250 μl digitonin wash buffer at 1.0 μg/mL final concentration for 1 h at 4 °C with rotation. Unbound pAG-MNase was removed by washing with digitonin wash buffer three times. Pre-chilled digitonin wash buffer containing 2 mM CaCl_2_ (200 μL) was added to the beads and incubated on ice for 30 min. The pAG-MNase digestion was halted by the addition of 200 μl 2× STOP solution (340 mM NaCl, 20 mM EDTA, 4 mM EGTA, 50 μg/ml RNase A, 50 μg/ml glycogen). The beads were incubated with shaking at 37 °C for 10 min in a tube shaker at 300 rpm to release digested DNA fragments from the insoluble nuclear chromatin. The supernatant was then collected by removing the magnetic beads. DNA in the supernatant was purified using the NucleoSpin Gel & PCR kit (Takara Bio, Kusatsu, Shiga, Japan). Sequencing libraries were then prepared from 3 ng DNA with the Kapa HyperPrep Kit (Roche) according to the manufacturer’s standard protocol. Libraries were multiplex sequenced (2 × 150 bp, paired-end) on an Illumina NovaSeq 6000 system to yield ~15 million mapped reads per sample. With separate replicated experiments, qPCR was used to examine enrichment at selected genomic regions. Primer sequences are provided in the **S1 Table.**

CUT&RUN sequence reads were processed with fastp [76] and aligned to the human GRCh38/hg38 reference genome assembly with Bowtie2 and yielding mapped reads in BAM files [77]. Hypergeometric Optimization of Motif EnRichment (HOMER) v4.11 was used for peak detection and their annotation utilizing the default parameters described in the developer’s manual [32]. Deeptools was used for making plotprofiles utilizing the default parameters [78]. Peaks and read alignments were visualized using the Integrated Genome Browser [79].

### Olink analysis

1.5 X 10^6^ THP-1 cells or vIL-6/THP-1 cells were prepared in triplicate and washed twice by PBS10ml and then suspended with 1ml fresh RPMI medium in 12 well plates. Inflammatory cytokines such as LPS (100ng/ml) and IFNα (100ng/ml) were then added to each well and cultured at 37°C, 5% CO_2_. 6 hours after incubation, supernatants were harvested and centrifuged (3,000 rpm, 3min) to remove the remaining cells. The resulting supernatant samples were stored at -20°C for approximately two weeks until they were sent for analysis. Samples wrapped in dry ice were then sent to Olink Proteomics (Temple City, CA). Olink’s Proximity Extension Assay (PEA) technology uses antibody pairs conjugated to unique oligonucleotides and is quantified via PCR. When both antibodies of a pair bind the target protein simultaneously, their respective conjugated oligonucleotides are brought into proximity, facilitating hybridization. The oligonucleotide sequence is then extended by DNA polymerase, amplified, and measured by qPCR to determine the sample’s initial protein abundance. Raw analyte expression values after PCR underwent multiple rounds of transformation by Olink, including a log2 transformation, and were returned as normalized protein expression (NPX) values [80]. For evaluating inflammatory cytokine production in the culture medium, the Olink Target 48 Cytokine panel was selected by multiple investigators. The data was processed with Olink Insight Stat Analysis software.

### RT-qPCR

vIL-6/THP-1, parental THP-1 cells and primary monocytes cells were infected with or without r219.KSHV for 72h and total RNA was extracted using the Quick-RNA miniprep kit (Zymo Research, Irvine, CA, USA). A total of 1 μg of RNA was incubated with DNase I for 15 minutes and reverse transcribed with the High Capacity cDNA Reverse Transcription Kit (Thermo Fisher, Waltham, MA USA). The resulting cDNA was used for qPCR. 10 μl SYBR Green Universal master mix (Bio-Rad) was used for qPCR according to the manufacturer’s instructions. Each sample was normalized to 18S ribosomal RNA, and the ddCt fold change method was used to calculate relative quantification. All reactions were run in triplicate. Primer sequences used for qRT-PCR are provided in the **S1 Table.**

### Immunoblotting

Cells were washed with PBS, lysed in lysis buffer (50 mM Tris-HCl [pH 6.8], 2% SDS, 10% glycerol) and boiled for 3min. The protein concentrations of the lysates were quantified with a BCA Protein Assay Kit (Thermo Fisher). Protein samples were separated by SDS-PAGE using 10% agarose gel and transferred to transfer membranes (Millipore-Sigma, St. Louis, MO, USA), which were incubated in 5% nonfat milk at room temperature for 2 hours. The membrane was incubated with the primary antibody at 4°C overnight or at room temperature for 2 hours. The membrane was then incubated with horseradish-peroxidase-conjugated secondary antibody or Alexa-647-conjugated secondary antibody at 25°C for 1 hour. For cell fractionation, cells were suspended with hypotonic buffer (20mM Tris-HCl (pH 7.4), 10mM NaCl, 3mM MgCl2, 0.5mM DTT and proteinase inhibitor cocktail) for 15min on ice and 0.5% NP-40 was added. Cells were then centrifuged for 10 minutes at 3000rpm to collect the supernatants (cytoplasm fraction). Pellets were washed with PBS 2 times and suspended with protein lysis buffer (nuclear fraction).

### Flow cytometry

Cells in culture were washed twice with PBS and resuspended in FACS buffer (PBS supplemented with 1% FBS) at 1x10^6^ cells/ml. Cells were then stained for 2 hours at room temperature with the gp130 antibodies (1:1000) followed by Alexa-647-conjugated secondary antibody for 1 hour. For intracellular nuclear staining to measure cell cycle, cells were washed twice with PBS and incubated with propidium iodide (PI) solution (50ng/ml PI, 0.2% NP-40, 0,25mg/ml RNase) for 15 min at 4°C and then for 30 min at 37°C in the dark. Flow cytometry was carried out by using a BD Acuri instrument (BD Biosciences) and data analysis was performed using FlowJo v10.8.1 (Tree Star) by gating on live cells based on forward versus side scatter profiles. The cell cycle was calculated by the Watson model implemented in FlowJo according to the manufacture’s protocol.

### Capture of nascent RNAs

To capture nascent RNAs, 0.4 mM EdU was added to the culture medium and was incorporated into the cells for 2 hours. Total RNA was prepared with an RNeasy Mini Kit (QIAGEN). The EdU-labeled RNAs were then biotinylated and captured by using the Click-it Nascent RNA Capture Kit (Life Technologies), in accordance with the manufacturer’s instructions. Briefly, 1 μg of EU-labeled RNA was biotinylated with 0.5 mM biotin azide in Click-iT reaction buffer. The biotinylated RNAs were precipitated with ethanol at 4°C overnight and resuspended in 50 μl distilled water. The biotinylated RNAs were then mixed with 12 μl Dynabeads MyOne Streptavidin T1 magnetic beads in Click-iT RNA binding buffer and heated at 68°C for 5 min, followed by incubation at room temperature for 30 min while gently vortexing. The beads were immobilized using the DynaMag-2 magnet and were washed with Click-iT wash buffers 1 and 2. The washed beads were resuspended in Click-iT wash buffer 2 and used for cDNA synthesis. cDNA synthesis was performed by the High Capacity cDNA Reverse Transcription Kit as described above.

### Statistical analysis

Experimental replicates of at least 3 for each sample, including negative controls, were prepared whenever applicable. Results are shown as mean ± SD from at least three independent experiments. Statistical analyses were performed using GraphPad Prism 9.4.1 software. Statistical significance was determined by appropriate testing with Student’s t-test and one-way ANOVA with Tukey’s multiple comparison test. A value of p < 0.05 was considered statistically significant.

## Supporting information

S1 Table List of Primer used for RT-qPCR and ChIP-qPCR. (XLSX)

S1 Fig hIL-6 is a functional homolog of vIL-6 (A) The number of up-regulated genes (log2 fold change >1, adj p-value < 0.01) after hIL-6 stimulation. Red circle represents THP-1 cells and green circle represents vIL-6/THP-1 cells. (B) Individual gene expression in THP-1 cells with vIL-6 stimulation (left, N = 8206) and hIL-6 stimulation (right, N = 8210). Representative gene names were labeled adjacent to dots. The red dashed line indicated log2 fold change = ±1 (vertical) and -log10 adj p-value = 2 (horizontal). (C) KEGG pathway analysis performed on up-regulated genes (log2 fold change >1, adj p-value < 0.01) in THP-1 cells with vIL-6 and hIL-6 stimulation. The result showed the top three pathways each. (D) Individual up-regulated gene expression (N = 348) in parent THP-1 and vIL-6/THP-1 cells after hIL-6 stimulation. Data were analyzed using Wilcoxon matched-pairs signed ranked test and shown as median. (E) Measurement of cell proliferation with MTT assays. 1 X 104 THP-1 or vIL-6/THP-1 cells were cultured in triplicate in a 96 well plate. vIL-6 was added to vIL-6/THP-1 cells every other day. OD (570-690nm) was measured on day 0,1,2,4 and 7. Data were analyzed using unpaired Student’s t test and shown as mean ± SD. (F) Immunoblotting with antibodies directed against STAT3, pshopho-STAT3 (Y705) and β-Actin (loading control) protein in THP-1 and vIL6/THP-1 cells. (G) FACS analysis showing the gp130 expression on cell surface. (H) The proportion of GFP-positive cells at 72 hours post-infection. The percentage was measured by flow cytometry.
(TIF)

S2 Fig **Prolonged vIL-6 exposure enhances the association of BRD4 and H3K27Ac (A)** BRD4 CUT &RUN signals in ±5kbp windows around the transcription start sites (TSS) of up-regulated genes in vIL-6/THP-1 cells (N = 303). **(B)** BRD4, H3K27Ac and H3K4me3 protein expression before and after vIL-6 stimulation in parental THP-1 and vIL-6/THP-1 cells. **(C)** DNA binding motif analysis of new BRD4 accumulation sites in vIL-6/THP-1 cells. Images were drawn by findMotif (HOMER). **(D)** The number of BRD4 and H3K27Ac peaks and their association in parental THP-1 and vIL-6/THP-1 cells. The overlapping peaks were extracted using mergepeaks (HOMER). **(E)** RNA pol II and H3K4me3 CUT &RUN signals in ±5kbp windows around the center of BRD4 and H3K27Ac peaks. **(F)** KEGG pathway analysis performed on genes at BRD4 and H3K27 overlapping peaks in promoter regions in vIL-6/THP-1 cells. Results are presented in descending order. **(G)** BRD4, RNA pol II, H3K27Ac and H3K4me3 enrichment in the *Cyclin E1* promoter region in parental THP-1 cells (pink) and vIL-6/THP-1 cells (blue). The peaks were visualized by importing the BAM files into the Integrative Genomics Viewer (IGV).
(TIF)

S3 Fig **Inflammatory response to IFNα after vIL-6 prolonged exposure (A)** hIL-6 production in THP-1 cells by LPS stimulation. THP-1 cells was incubated with LPS 100ng/ml or 1μg/ml for various time periods. Supernatants were harvested and incubated in triplicate in ELISA plate coated with hIL-6 antibody, Human IL-6 Uncoated ELISA kit (Invitrogen) was then used to evaluate the hIL-6 production by following the manufacturer’s guideline. The protein binding measured as OD values at 450nm was shown. Results are presented as mean percentage viability ±SD (*n*  =  3 samples/group). Data was analyzed by a one-way ANOVA test. **(B)** Heatmap showing the results of Olink Target 48 Cytokine panel. IFNα (100ng/ml) was added to parent THP-1 cells or vil-6/THP-1 cells for 6 hours. Cytokine production in untreated THP-1 cells was set as 1 and log_2_ fold activation relative to untreated cells are shown. Samples were prepared in triplicate and the mean value were shown. **(C)** Inflammatory cytokine production determined by Olink proximity extension assay. Data was analyzed using two-sided unpaired Student’s *t* test and shown as mean ± SD.
(TIF)

S4 Fig **BRD4 enrichment in the promoter region of inflammatory genes (A)** BRD4, RNA pol II, H3K27Ac and H3K4me3 enrichment in hIL-6, IL-10, IL-1β promoter region in parental THP-1 cells (pink) and vIL-6/THP-1 cells (blue). The promoter region is enclosed by a black line. Each CUT&RUN peak was visualized by importing the BAM files into Integrative Genomics Viewer (IGV). **(B)** Schematic diagram of nascent RNA labeling after LPS stimulation. LPS were added to culture medium in parental THP-1 and vIL-6/THP-1 cells and cells were incubated with EU for 1 hour at 0, 1, 2, 4 8, 12 h post LPS stimulation. **(C)** Immunoblotting of BRD4, BRD2 and β-Actin protein in THP-1 cells with or without BRD4 inhibitors.
(TIF)
