# Supplementary figures and images for: KSHV vIL-6 enhances inflammatory responses by epigenetic reprogramming
Source: PLoS Pathog. 2023 Nov 7;19(11):e1011771. doi: 10.1371/journal.ppat.1011771 (PMC10656005; doi:10.1371/journal.ppat.1011771)

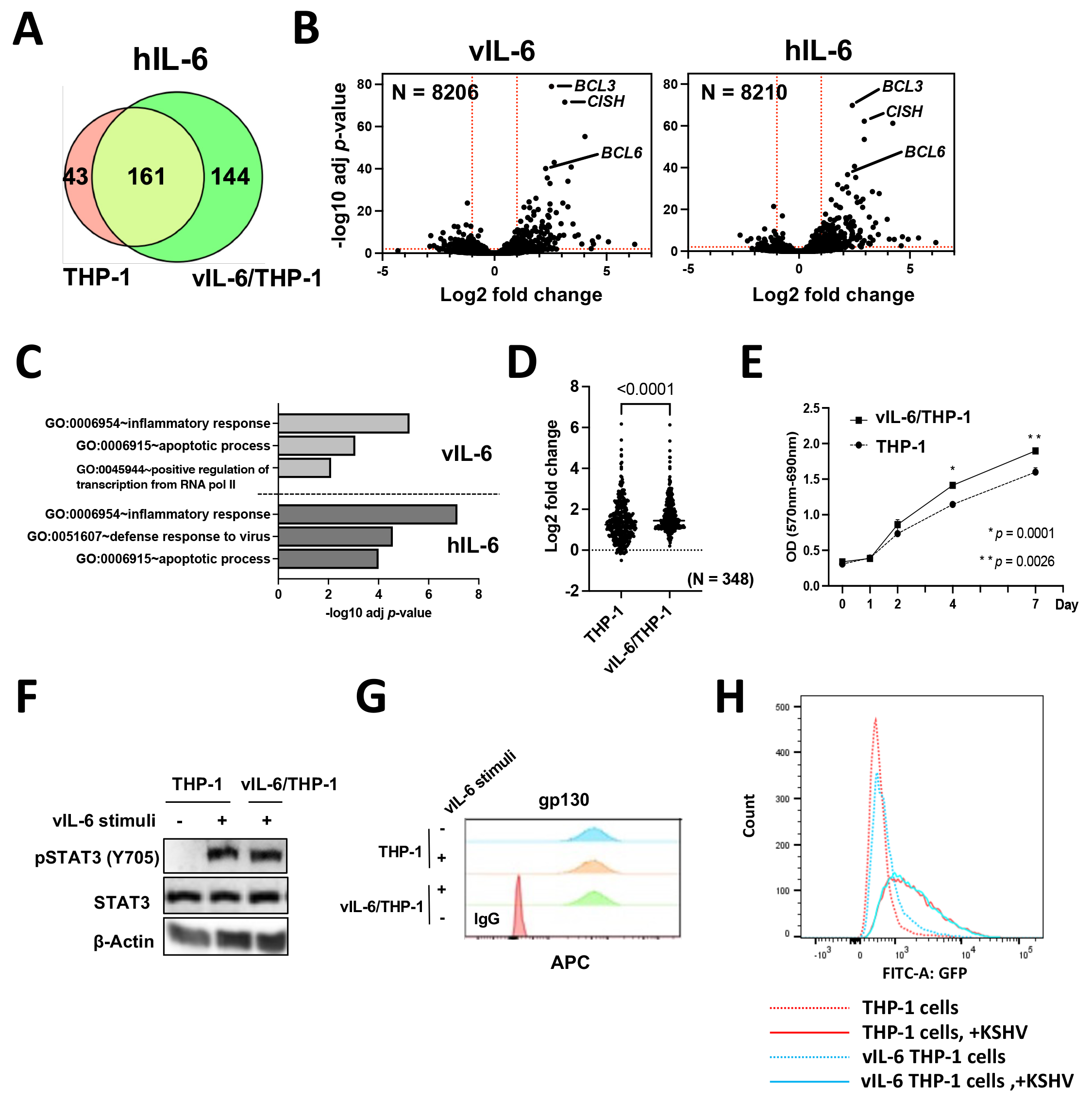

Supplement: S1 Fig — hIL-6 is a functional homolog of vIL-6 (A) The number of up-regulated genes (log2 fold change >1, adj p-value < 0.01) after hIL-6 stimulation. Red circle represents THP-1 cells and green circle represents vIL-6/THP-1 cells. (B) Individual gene expression in THP-1 cells with vIL-6 stimulation (left, N = 8206) and hIL-6 stimulation (right, N = 8210). Representative gene names were labeled adjacent to dots. The red dashed line indicated log2 fold change = ±1 (vertical) and -log10 adj p-value = 2 (horizontal). (C) KEGG pathway analysis performed on up-regulated genes (log2 fold change >1, adj p-value < 0.01) in THP-1 cells with vIL-6 and hIL-6 stimulation. The result showed the top three pathways each. (D) Individual up-regulated gene expression (N = 348) in parent THP-1 and vIL-6/THP-1 cells after hIL-6 stimulation. Data were analyzed using Wilcoxon matched-pairs signed ranked test and shown as median. (E) Measurement of cell proliferation with MTT assays. 1 X 104 THP-1 or vIL-6/THP-1 cells were cultured in triplicate in a 96 well plate. vIL-6 was added to vIL-6/THP-1 cells every other day. OD (570-690nm) was measured on day 0,1,2,4 and 7. Data were analyzed using unpaired Student’s t test and shown as mean ± SD. (F) Immunoblotting with antibodies directed against STAT3, pshopho-STAT3 (Y705) and β-Actin (loading control) protein in THP-1 and vIL6/THP-1 cells. (G) FACS analysis showing the gp130 expression on cell surface. (H) The proportion of GFP-positive cells at 72 hours post-infection. The percentage was measured by flow cytometry. (TIF) [file ppat.1011771.s002.tif]

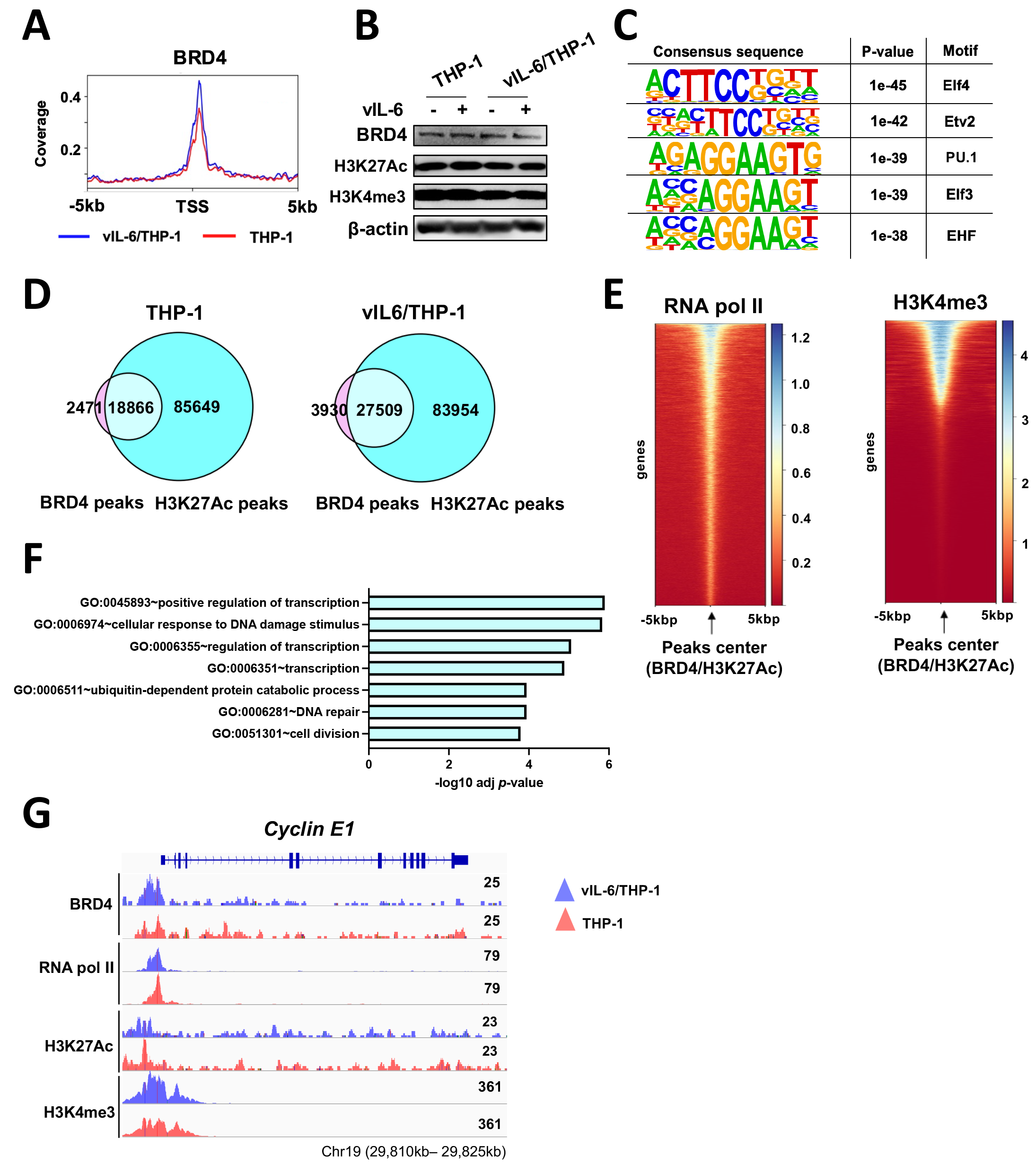

Supplement: S2 Fig — Prolonged vIL-6 exposure enhances the association of BRD4 and H3K27Ac (A) BRD4 CUT &RUN signals in ±5kbp windows around the transcription start sites (TSS) of up-regulated genes in vIL-6/THP-1 cells (N = 303). (B) BRD4, H3K27Ac and H3K4me3 protein expression before and after vIL-6 stimulation in parental THP-1 and vIL-6/THP-1 cells. (C) DNA binding motif analysis of new BRD4 accumulation sites in vIL-6/THP-1 cells. Images were drawn by findMotif (HOMER). (D) The number of BRD4 and H3K27Ac peaks and their association in parental THP-1 and vIL-6/THP-1 cells. The overlapping peaks were extracted using mergepeaks (HOMER). (E) RNA pol II and H3K4me3 CUT &RUN signals in ±5kbp windows around the center of BRD4 and H3K27Ac peaks. (F) KEGG pathway analysis performed on genes at BRD4 and H3K27 overlapping peaks in promoter regions in vIL-6/THP-1 cells. Results are presented in descending order. (G) BRD4, RNA pol II, H3K27Ac and H3K4me3 enrichment in the Cyclin E1 promoter region in parental THP-1 cells (pink) and vIL-6/THP-1 cells (blue). The peaks were visualized by importing the BAM files into the Integrative Genomics Viewer (IGV). (TIF) [file ppat.1011771.s003.tif]

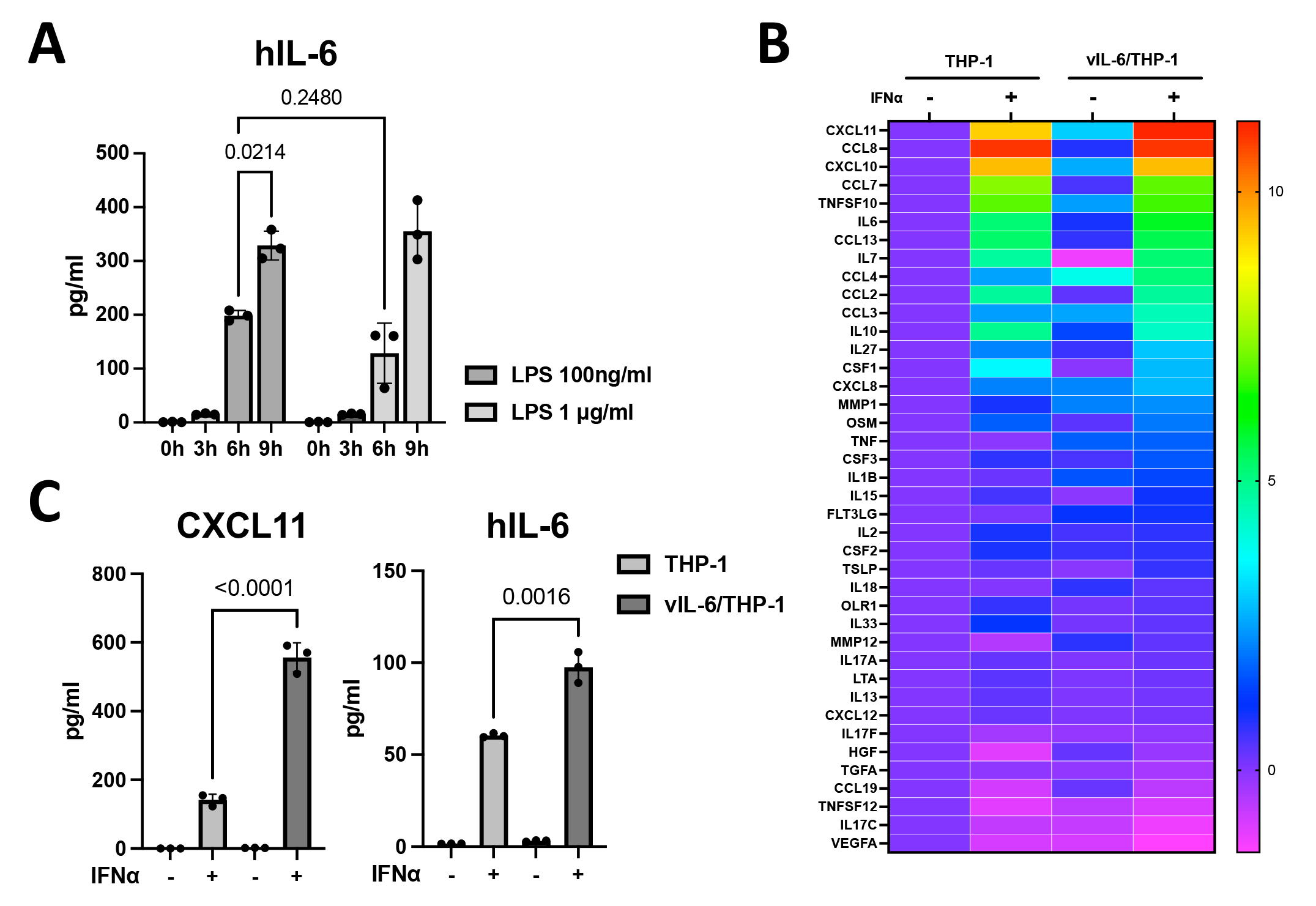

Supplement: S3 Fig — Inflammatory response to IFNα after vIL-6 prolonged exposure (A) hIL-6 production in THP-1 cells by LPS stimulation. THP-1 cells was incubated with LPS 100ng/ml or 1μg/ml for various time periods. Supernatants were harvested and incubated in triplicate in ELISA plate coated with hIL-6 antibody, Human IL-6 Uncoated ELISA kit (Invitrogen) was then used to evaluate the hIL-6 production by following the manufacturer’s guideline. The protein binding measured as OD values at 450nm was shown. Results are presented as mean percentage viability ±SD (n = 3 samples/group). Data was analyzed by a one-way ANOVA test. (B) Heatmap showing the results of Olink Target 48 Cytokine panel. IFNα (100ng/ml) was added to parent THP-1 cells or vil-6/THP-1 cells for 6 hours. Cytokine production in untreated THP-1 cells was set as 1 and log2 fold activation relative to untreated cells are shown. Samples were prepared in triplicate and the mean value were shown. (C) Inflammatory cytokine production determined by Olink proximity extension assay. Data was analyzed using two-sided unpaired Student’s t test and shown as mean ± SD. (TIF) [file ppat.1011771.s004.tif]

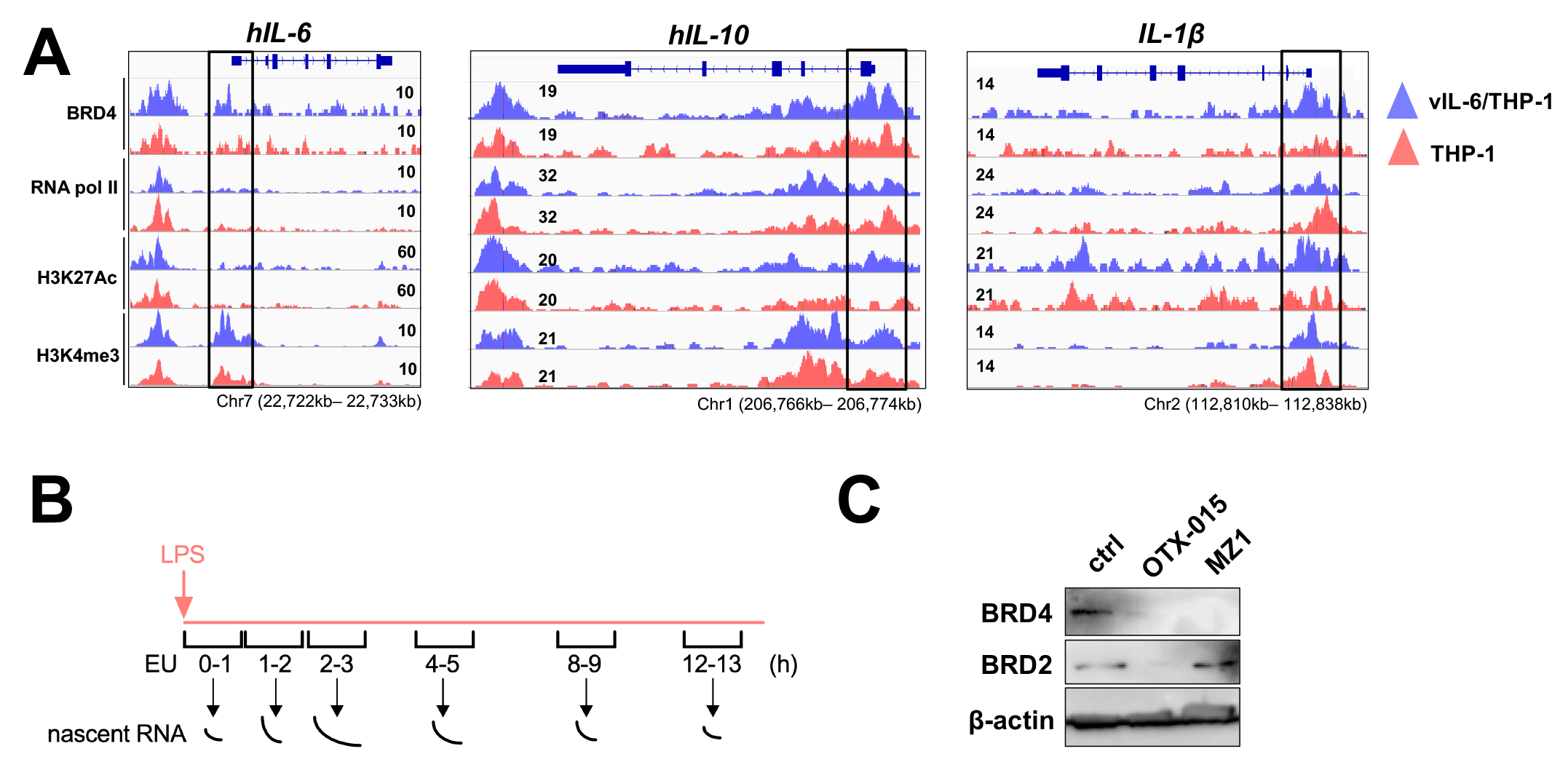

Supplement: S4 Fig — BRD4 enrichment in the promoter region of inflammatory genes (A) BRD4, RNA pol II, H3K27Ac and H3K4me3 enrichment in hIL-6, IL-10, IL-1β promoter region in parental THP-1 cells (pink) and vIL-6/THP-1 cells (blue). The promoter region is enclosed by a black line. Each CUT&RUN peak was visualized by importing the BAM files into Integrative Genomics Viewer (IGV). (B) Schematic diagram of nascent RNA labeling after LPS stimulation. LPS were added to culture medium in parental THP-1 and vIL-6/THP-1 cells and cells were incubated with EU for 1 hour at 0, 1, 2, 4 8, 12 h post LPS stimulation. (C) Immunoblotting of BRD4, BRD2 and β-Actin protein in THP-1 cells with or without BRD4 inhibitors. (TIF) [file ppat.1011771.s005.tif]
